# Validity and reliability of the Spanish version of the Epworth Sleepiness Scale

**DOI:** 10.1007/s11325-025-03339-7

**Published:** 2025-05-17

**Authors:** Adalberto Campo-Arias, Edwin Herazo, John Carlos Pedrozo-Pupo

**Affiliations:** 1 Universidad del Magdalena, Calle 29H3 No. 22-01, Santa Marta, 470004 Colombia; 2 Instituto de Investigación del Comportamiento Humano, 110221 Bogotá, Colombia

**Keywords:** Sleepiness, Reliability, Validity, University students, Validation studies

## Abstract

**Background:**

The Epworth Sleepiness Scale (ESS) is a brief instrument to identify sleep propensity. However, little is known about the psychometric performance of the Spanish version in university students. The study aimed to study the validity and reliability of the ESS in Colombian university students.

**Methods:**

A psychometric study was designed with 465 students of health-related careers between 18 and 29 years old (*M* = 20.48, *SD* = 2.27); 66.67% of the students were women. A confirmatory factor analysis was conducted, comparisons of scores between men and women, correlations with insomnia (Athens Insomnia Scale, AIS), anxiety (GAD-7), depression (PHQ-9), and sleep hygiene (SHI-10) and sex differential item functioning as indicators of validity and Cronbach’s alpha and McDonald’s omega were calculated as estimators of internal consistency.

**Results:**

The ESS showed a unidimensional structure, similar scores in men and women, statistically significant correlations with AIS, GAD-7, PHQ-9, and SHI-10, without sex differential item functioning, and high internal consistency (Cronabch’s alpha and McDonald’s omega of 0.82).

**Conclusion:**

The Spanish version of the ESS presents acceptable validity and reliability indicators in Colombian university students. However, these findings must be corroborated in other samples of Spanish-speaking participants.

## Introduction

Murray W. Johns designed the Epworth Sleepiness Scale (ESS) for use as a rapid screening measure in people with suspected obstructive sleep apnea and found that scores on the ESS reflected a propensity to sleep that was positively related to apnea severity and, conversely, people with insomnia showed low scores on the measures [1]. However, the ESS has a low specificity for obstructive sleep apnea [2].

The ESS measures sleepiness in some specific situations without implying the cause rather than the “propensity to sleep” due to the limited definition of the latter concept [3]. Sleepiness measured by the ESS may be present in other sleep disorders, some mental disorders such as depression, and even in people who sleep a few hours due to work overload [4, 5].

Psychometric performance often varies depending on the respondents’ cultural and social characteristics [6]. In the clinical context and healthy controls, the ESS has shown acceptable psychometric performance in patients with different clinical situations, generally with a unidimensional structure, high internal consistency, and significant correlations with other instruments that measure related constructs [7].

However, information on the validity and reliability indicators of the ESS outside the clinical context is minimal [8–16]. ESS has shown usefulness in the general population [8–11]. Rosales-Mayor et al. [8], the EES showed a two-dimensional structure in 219 Peruvian adults between 18 and 65 years old, with a Cronbach’s alpha for the global scale of 0.79. Nariya et al. [9], In 80 people aged 18–60 in India, they observed high internal consistency (Cronbach’s alpha of 0.97). Gunawardane [10], in 283 heavy vehicle drivers in Sri Lanka, confirmed the two-dimensional structure of the ESS and reported that the overall scale had acceptable internal consistency, Cronbach’s alpha of 0.74. Oneib et al. [11], among 120 asymptomatic adults in Morocco, documented acceptable internal consistency, Cronbach’s alpha of 0.71, and significantly higher scores in women than in men (*p* < 0.05). However, Fernandes et al. [12], In 1,016 adults from the general community in Brazil, observed that the EES had low correlations with the Pittsburgh Sleep Quality Index (*r*_*s*_ = 0.03) and Insomnia Severity Index (*r*_*s*_ = 0.08).

Also, in undergraduate students, Gelaye et al. [13], among 8,481 university students from Chile, Ethiopia, Peru, and Thailand, documented that the ESS showed a two-dimensional structure with acceptable goodness-of-fit indicators, high internal consistency (with omission of Cronbach’s alpha value) and significant correlation (*r* = 0.71) with the Pittsburgh Sleep Quality Index. Pilcher et al. [14], in 292 students from a university in Austria and 329 from a university in the United States, in both samples, the ESS presented a two-dimensional structure with acceptable internal consistency, with Cronbach’s alpha of 0.75 and 0.72 for the eight items (they omitted Cronbach’s alpha for each dimension). Manzar et al. [15], among 600 students in Ethiopia, observed that the one-dimensional structure adequately fit the data, with Cronbach’s alpha of 0.75. Attal et al. [16] observed a two-dimensional structure in 180 medical students in Yemen. However, the factors showed low internal consistency: factor 1 (sitting and lying), Cronbach’s alpha of 0.65, and factor 2 (interactive situations), 0.62.

In Colombia, Chica-Urzola et al. [17] observed that the ESS presented high internal consistency with Cronbach’s alpha of 0.85 in 150 patients with sleep disorders. Pedrozo-Pupo et al. [18], in 684 patients referred for evaluation by polysomnography, found that the ESS showed a unidimensional structure and high consistency with Cronbach’s alpha and McDonald’s omega of 0.80.

The studies reviewed in previous paragraphs showed that the psychometric performance of the ESS in the general population and university students has been more variable than that reported in populations that consult for some sleep abnormality [8–18]. According to recent meta-analyses, sleepiness can affect a high percentage of university students in the health area, with a pooled prevalence between 33 and 43% [19–21]. Sleepiness is more likely related to lifestyle or negative emotional states in young adult students than obstructive sleep apnea [22, 23]. The ESS is an instrument with high sensitivity and low specificity to identify sleep propensity [2].

Consequently, it is crucial to know the psychometric performance of health measurement instruments in different settings to ensure the validity and reliability of the findings at other times or populations. Likewise, this process helps in the understanding and precision of the concepts, which invites the refinement of the instrument for future measurements [7]. In the present study, a confirmatory factor analysis is performed to specify the dimensionality of the ESS in Spanish. In addition, the nomological validity or nomothetic span of the instrument is expanded with other measurement scales rarely reported in university students; two coefficients are calculated for the internal consistency of the instrument to guarantee this indicator of reliability and, finally, the differential functioning of the items according to sex is explored for the first time. In the university setting, it is relevant to quickly and easily identify, validly and reliably, cases of sleepiness. Identifying the cause of the symptoms and the appropriate management are essential since sleepiness can negatively affect students’ academic performance and quality of life [22].

The objective of the present study was to study the reliability (internal consistency) and validity (dimensionality, nomothetic span, and sex differential item functioning [sex-DIF]) of the ESS in Colombian university students.

## Materials and methods

### Design

The current research is a validation or test evaluation study that uses intuitive statistical tests to explore the dimensionality, nomothetic span, and sex-DIF of the instrument’s response pattern [6].

### Participants

The sample consisted of 465 emerging adult students aged 18–29 (*M* = 20.48, *SD* = 2.27), students of health sector courses: dentistry, medicine, nursing, and psychology. This sample was adequate for a confirmatory factor analysis; generally, 400 participants are sufficient if most loadings are above 0.40 [24]. Table 1 provides more information on the students’ demographic descriptions.

**Table 1:** Demographic description of participating students

Variable	Frequency	%
Age (years)		
18–20 21–29	276 189	59.53 40.47
Sex		
Female Male	310 155	66.67 33.33
Career		
Dentistry Nursing Medicine Psychology	111 124 129 111	23.23 25.80 27.74 23.23
Semester		
Basic or introductory Advanced or clinical	241 224	51.83 48.17
Origin		
Urban Rural	385 80	82.79 17.21
Family income		
Low Middle or high	330 135	70.97 29.03

## Tools

### ESS

The ESS is an eight-item instrument that quantifies the likelihood of falling asleep in eight everyday situations. It offers four response options, which are scored between zero and three. Total scores range from 0 to 24, with higher scores indicating a higher risk of sleepiness [1, 25]. The present study used a Spanish version revised and analyzed by other Colombian authors [18].

### Athens insomnia scale (AIS)

The AIS consists of eight items that quantify insomnia. The first five items explore nighttime sleep; the remaining three explore daytime insomnia-related discomfort. Each item provides four response options, scoring from zero to three, with a higher score indicating a greater risk of insomnia [26]. The present study’s AIS showed high internal consistency, with Cronbach’s alpha of 0.81.

### General anxiety disorder scale (GAD-7)

The GAD-7 includes seven items that assess anxiety symptoms and presents four response options scored from zero to three; the higher the score, the greater the risk of anxiety with clinical significance [27]. The GAD-7 showed adequate internal consistency in the present research sample, with Cronbach’s alpha of 0.90.

### Patient health questionnaire (PHQ-9)

The PHQ-9 screens symptoms with nine questions and four response options, to which scores range from zero to three; the higher the score, the greater the risk of depression [28]. The internal consistency of the PHQ-9 was high, with Cronbach’s alpha 0.88 in the present investigation.

### Sleep hygiene inventory (SHI-10)

The SHI-10 consists of ten items exploring behaviours affecting sleep quantity and quality. Five response options are scored from zero to four, with total scores ranging from 0 to 40; the higher the score, the worse the sleep hygiene [29]. The SHI-10 showed acceptable internal consistency in the present student sample, with Cronbach’s alpha of 0.76.

### Procedure

Data collection was conducted in the second academic semester of 2022 and 2023. In the classroom, through group application, students were given an online questionnaire that included informed consent, demographic information, and the instruments detailed above. A research assistant explained the study’s objectives and that participation was voluntary. The questionnaire with the items was only deployed to students who consented to participate.

### Statistical analysis

#### Validity

##### Dimensionality

Initially, exploratory factor analysis (EFA) was performed in which the Kaiser-Meyer-Olkin measure of sampling adequacy (KMO), Bartlett’s sphericity test, Eigenvalue, and total variance explained tests were calculated. The dimensionality of the ESS-10 was verified by confirmatory factor analysis (CFA). The loadings for each item were found, and the goodness-of-fit indicators were calculated to confirm that they fit the proposed model: chi-square and standardized chi-square, root mean square error of approximation (*RMSEA*) with 90% confidence interval (*90%CI*), comparative fit index (*CFI*), Tucker-Lewis Index (*TLI*) and standardized root mean square residual (*SRMR*). The CFA confirms the dimensionality when the standardized chi-square is less than 3.00, the *RMSEA* is less than 0.06, the *CFI* and *TLI* are greater than 0.90, and the *SRMR* is less than 0.05 [30].

### Nomothetic span or nomological validity

The nomothetic span was estimated using several statistical tests. The comparison of scores between women and men was performed with the unpaired Student *t*-test. The authors hypothesized that men and women would present similar scores based on a meta-regression [19]. They also estimated Pearson or Spearman correlations based on the data distribution of the total scores of the ESS, AIS, GAD-7, PHQ-9, and SHI-10. Nomological validity was accepted if Pearson (*r*) or Spearman (*r*_*s*_) correlations equal to or greater than 0.30 were observed, with a probability value less than 0.01 [24]. The Shapiro-Francia test was used to demonstrate the symmetric distribution of scores.

### Sex-DIF

The sex-DIF was estimated with Kendall’s tau-b correlation coefficient, which is the best option when one of the variables analyzed is dichotomous. To achieve this, one represented the female sex, and zero represented the male sex. The sex-DIF acceptance criterion was taken if the correlations between sex and item were higher than 0.20 [24].

### Reliability

Internal consistency was determined by calculating Cronbach’s alpha and McDonald’s omega coefficients. McDonald’s omega is a better indicator of internal consistency when the tau equivalence principle is violated, an assumption necessary for calculating Cronbach’s alpha; that is when the factor loadings of the items show similar values. The description of the items was completed by calculating the mean (*M*), standard deviation (*SD*), corrected correlation between the item and the total score, and Cronbach’s alpha if the item was omitted. The statistical analysis was completed using IBM-SPSS version 23.0, STATA version 17.0, and Jamovi version 2.3.

### Ethical aspects

The students signed an informed consent form online. Measurement scales were used free of charge following national and international standards for research on human subjects by the ethical standards of the institutional and/or national research committee (The ethics research committee of the Universidad del Magdalena reviewed and approved the research project according to minutes 005 of the ordinary virtual session of June 9, 2022, and second, 007 of the ordinary virtual session of August 31, 2023), along with the 1964 Helsinki Declaration and its later amendments or comparable ethical standards.

### Data availability

The corresponding author can request data supporting this research with reasonable justification.

## Results

The EFA showed a KMO of 0.83 and Bartlett’s sphericity test of 1,055.33 (*df* = 28, *p* < 0.001). One factor was retained, presenting an Eigenvalue of 3.55, and explained 44.36% of the total variance. The unidimensional structure of the ESS was confirmed, which showed excellent goodness-of-fit indicators. The ESS loadings were higher than 0.40, between 0.43 (item 5, ‘lying down and resting in the afternoon’) and 0.67 (items 3, ‘sitting and inactive in a public place,’ and 6, ‘sitting and talking to someone’). Table 2 presents loadings of items.

**Table 2:** Factor loadings of the ESS items

Item/factor	Loading
1. Sitting and reading	0.62
2. Watching television	0.55
3. Sitting, inactive in a public place	0.67
4. As a passenger in a car for an hour without a break	0.61
5. Lying down to rest in the afternoon when circumstances permit	0.43
6. Sitting and talking to someone	0.67
7. Sitting quietly after lunch without alcohol	0.62
8. In a car, while stopped for a few minutes in the traffic	0.66

The parsimonious model of the one-dimensionality of the ESS was unsatisfactory; the data did not fit the model: Chi-square = 158.03, *df* = 20, *p* < 0.001, normalized chi-square = 7.90, *RMSEA* = 0.12, *90%CI* 0.11–0.15, *CFI* = 0.87, *TLI* = 0.81, and *SRMR* = 0.06. Given these unacceptable values of the goodness-of-fit indicators, modification indices were applied with the inclusion of the covariance between items 3 and 6, 4 and 6, 4 and 8, and 5 and 7. This inclusion of covariances significantly improved the unidimensional model: Chi-square = 46.26, *df* = 16, *p* < 0.001; normalized chi-square = 2.89, *RMSEA* = 0.06, *90%CI* 0.04–0.09, *CFI* = 0.97, *TLI* = 0.94, and *SRMR* = 0.03.

The nomological validity of the ESS was explored: women showed higher scores than men [*M* = 12.95 (*SD* = 4.77) versus *M* = 10.06 (*SD* = 4.61); Levene’s test of homogeneity of variance *F* = 0.15, *p* = 0.70, *t* = 6.23, *df* = 463, *p* = 0.001]. The total score of the ESS (Shapiro-France test *V’* = 0.77, *p* = 0.72) and the AIS showed a symmetrical distribution (Shapiro-France test *V’* = 0.81, *p* = 0.67). However, the distribution was skewed for GAD-7 (Shapiro-France test *V’* = 9.76, *p* < 0.01) and PHQ-9 (Shapiro-France test *V’* = 11.40, *p* < 0.01). Correlations were significant with total anxiety scores (GAD-7, *r*_*s*_ = 0.38), depression (PHQ-9, *r*_*s*_ = 0.48), sleep hygiene (SHI-10, *r* = 0.60), and insomnia (AIS, *r* = 0.46).

Similarly, the items showed similar sex-DIF with Kendall’s tau-b correlations less than 0.20, except for item 4 (‘As a passenger in a car for one hour of continuous driving’). Table 3 shows all Kendall’s tau-b correlations.

**Table 3:** Kendall’s Tau B according to sex

Item	Kendall
1. Sitting and reading	0.14
2. Watching television	0.19
3. Sitting, inactive in a public place	0.13
4. As a passenger in a car for an hour without a break	0.23
5. Lying down to rest in the afternoon when circumstances permit	0.16
6. Sitting and talking to someone	0.15
7. Sitting quietly after lunch without alcohol	0.15
8. In a car, while stopped for a few minutes in the traffic	0.19

Scores on the ESS ranged from 0 to 24 (*M* = 11.99, *SD* = 4.91). Cronbach’s alpha and McDonald’s omega were 0.82. Table 4 reports the means, standard deviations, corrected correlations between the item and the total score, and Cronbach’s alphas if the item was omitted.

**Table 4:** Description of ESS items

Item	M	SD	CC	Alpha*	Omega*
1. Sitting and reading	1.62	0.93	0.55	0.80	0.80
2. Watching television	1.42	0.98	0.50	0.80	0.81
3. Sitting, inactive in a public place	1.10	0.95	0.57	0.79	0.79
4. As a passenger in a car for an hour without a break	1.86	0.99	0.56	0.79	0.80
5. Lying down to rest in the afternoon when circumstances permit	2.38	0.79	0.40	0.81	0.82
6. Sitting and talking to someone	0.64	0.74	0.57	0.80	0.80
7. Sitting quietly after lunch without alcohol	1.86	0.95	0.57	0.79	0.80
8. In a car, while stopped for a few minutes in the traffic	1.11	1.04	0.58	0.79	0.79

Note: *M* = mean. *SD* = standard deviation. CC = corrected correlation between the item and the total score. *If the item was omitted

Since item 4 showed biased behaviour according to sex, possible calculations were performed stratified by sex. Among females, the one-dimensionality with inclusion of the covariance between items 3 and 6, 4 and 6, 4 and 8, and 5 and 7 was satisfactory: Chi-square = 43.26, *df* = 16, *p* < 0.001; normalized chi-square = 2.69, *RMSEA* = 0.07, *90%CI* 0.05–0.10, *CFI* = 0.96, *TLI* = 0.93, and *SRMR* = 0.04. Correlations were significant with total anxiety scores (*r*_*s*_ = 0.37), depression (*r*_*s*_ = 0.49), sleep hygiene (*r* = 0.60), and insomnia ( *r* = 0.46). Finally, Cronbach’s alpha was 0.80, and McDonald’s omega was 0.81.

Among males, the one-dimensionality with inclusion of the covariance between items 3 and 5, 3 and 6, 4 and 6, and 6 and 8 was acceptable: Chi-square = 36.02, *df* = 16, *p* < 0.001; normalized chi-square = 2.25, *RMSEA* = 0.09, *90%CI* 0.05–0.13, *CFI* = 0.93, *TLI* = 0.88, and *SRMR* = 0.05. Correlations were statistically significant with total anxiety scores (*r*_*s*_ = 0.34), depression (*r*_*s*_ = 0.38), sleep hygiene (*r* = 0.52), and insomnia (AIS, *r* = 0.45). Finally, Cronbach’s alpha and McDonald’s omega were 0.81.

## Discussion

The present study shows that the Spanish version of the ESS has acceptable validity and reliability indicators in Colombian university students. The ESS presents a unidimensional structure, similar scores in men and women, statistically significant correlations with other theoretically and empirically related constructs, and high internal consistency.

The one-dimensionality of the ESS observed in the present study is consistent with that documented in university students in Ethiopia [15]. However, other research noted that a two-dimensional solution best fit the data of the students analyzed [13, 14, 16], with the limitation that the only study that reported the internal consistency of each dimension was below 0.70 (16).

Regarding the nomothetic span in the present study, it was found that the ESS maintained significant correlations with other sleep-related measures (insomnia and sleep hygiene) and emotional symptoms that may be accompanied by sleep pattern disturbances (anxiety and depression). However, the scores were similar in the entire sample, with no difference by sex, as initially hypothesized. This observation converges with the findings in a large sample of university students from three continents in which the ESS showed a high correlation with the Pittsburgh Sleep Quality Index [13]. Other psychometric investigations with university students omitted the comparison with AIS, GAD-7, PHQ-9, and SHI-10, and additionally, the comparison of scores by sex and the differential functioning of items was not explored.

The ESS showed excellent internal consistency in the present sample of emerging adult students. This data is consistent with other studies that showed that the one-dimensional structure of the ESS achieved internal consistency in the recommended range between 0.70 and 0.90, with values between 0.72 and 0.75 [14, 15].

These divergent validity and reliability indicators are frequent in psychometric studies and are related to the participants’ demographic, cultural, and clinical characteristics. This situation may explain the better overall performance of the ESS in clinical populations with a high frequency and greater severity of sleepiness, regardless of the cause, than in the general population and university students. These findings highlight the need to repeat the psychometric evaluation in each new population [6].

It is essential to identify sleepiness quickly and accurately in the university environment; evidence of the validity and reliability of the available tools is necessary [6]. Understanding and addressing its root causes is essential, as sleepiness can negatively affect students’ academic performance and general well-being [2].

This study had the strength of thoroughly exploring the nomological validity of the ESS with five different comparisons, showing for the first time that the ESS items are free of sex bias, except for item 4 (‘As a passenger in a car for one hour of continuous driving’) and calculating two internal consistency coefficients. However, it had the limitation that it accepted the null hypothesis for the scores in men and women. Future research should explore whether this difference may be mediated by cultural or linguistic factors that are impossible to identify without cross-cultural or cross-linguistic comparison [6].

It is concluded that the Spanish version of the ESS presents acceptable validity and reliability among Colombian university students. However, other samples of Spanish-speaking participants must be corroborated to corroborate these findings.
